# Formative research to support the implementation of respondent-driven sampling in a national study on HPV among vulnerable populations

**DOI:** 10.1590/0034-7167-2024-0581

**Published:** 2025-12-08

**Authors:** Giovana Petracco de Miranda, Camila Bonalume Dall’ Aqua, Tássia Rolim Camargo, Natália Luiza Kops, Eliana Márcia Wendland

**Affiliations:** I Hospital Moinhos de Vento. Porto Alegre, Rio Grande do Sul, Brazil; II Universidade Federal de Ciências da Saúde de Porto Alegre. Porto Alegre, Rio Grande do Sul, Brazil

**Keywords:** Sexual Health, Sexual and Gender Minorities, Vulnerable Populations, Human Papillomavirus, Method., Salud Sexual, Minorías Sexuales y de Género, Poblaciones Vulnerables, Virus del Papiloma Humano, Método.

## Abstract

**Objectives:**

to describe the formative research carried out during the implementation of respondent driven sampling to assess human papillomavirus prevalence among gay men, men who have sex with men, and female sex workers in Brazil.

**Methods:**

meetings were held between the study team, healthcare professionals, and representatives of social movements. Two capital cities from each region of Brazil were included between 2018 and 2021, totaling 11 meetings.

**Results:**

the discussions addressed logistical feasibility, the definition of incentives and benefits, the selection of seeds, and expectations regarding recruitment.

**Final Considerations:**

formative research is essential for developing studies that use the respondent-driven sampling method, as the involvement of target population representatives supports the collection of information on their specific characteristics.

## RESUMEN

**Objetivos:**

describir la investigación formativa realizada para la implementación del método *Respondent Driven Sampling* con el fin de evaluar la prevalencia del virus del papiloma humano en *gays*, hombres que tienen sexo con hombres y trabajadoras sexuales en Brasil.

**Métodos:**

se llevaron a cabo 11 encuentros entre 2018 y 2021 en dos capitales de cada región del país, involucrando al equipo de investigación, profesionales de la salud y representantes de movimientos sociales.

**Resultados:**

se abordaron temas como adecuaciones logísticas, definición de incentivos primario y secundario, selección de semillas y expectativas respecto al reclutamiento.

**Consideraciones Finales:**

la investigación formativa es fundamental para desarrollar estudios que emplean el método RDS, ya que la participación de representantes de poblaciones clave contribuye a la recolección de información específica sobre sus particularidades.

## INTRODUCTION

According to data from the Global Burden of Disease (GBD), 8.74 million people were living with HIV worldwide in 1990^(1)^, rising significantly to 39.9 million in 2023^(2)^. The HIV epidemic brought increased attention to certain groups within public health, particularly individuals in situations of vulnerability who are at greater risk for specific diseases due to social determinants of health. These groups came to be known as key populations or vulnerable populations. They include gay men and other men who have sex with men (MSM), people who use alcohol and other drugs, female sex workers, transgender women and travestis, and people deprived of liberty^(3)^.

These populations are disproportionately affected by certain conditions, especially sexually transmitted infections (STIs), due to barriers to accessing prevention, testing, and treatment^(3)^. Among these infections is human papillomavirus (HPV), which, in addition to causing warty lesions, is associated with the development of various types of cancer and is linked to the same behaviors and practices that increase vulnerability to other STIs^(4)^.

Because these groups are widely recognized as hard-to-reach populations, specific sampling methods are needed for scientific research. One such method is respondent-driven sampling (RDS), developed in the 1990s based on snowball sampling^(5)^. Key features of RDS include the selection of seeds-individuals with strong social networks who initiate the study-and peer recruitment, in which each participant receives coupons to invite others from the same population. Due to its effectiveness and its ability to produce adjusted and generalizable estimates, RDS has become widely used in studies in Brazil^(6,7)^ and other countries^(8-11)^.

To ensure successful implementation of RDS, formative research must be conducted to gather information about the population and its specific characteristics and to engage its members in the planning and organization of the study at the local level. Formative research takes place before data collection begins and is a crucial step in adapting the study to the population’s needs, encouraging their involvement, and facilitating its implementation.

## OBJECTIVES

To describe the formative research carried out during the implementation of respondent driven sampling to assess human papillomavirus prevalence among gay men, men who have sex with men, and female sex workers in Brazil.

## METHODS

This study aimed to understand the social organization of gay men/MSM and female sex workers in the cities included in the SMESH study, to support the implementation of the RDS method. SMESH is a nationwide cross-sectional study designed to assess the prevalence of HPV and other STIs among gay men/MSM and female sex workers of all biological sexes. Ten capital cities across Brazil’s five regions were included: Boa Vista, Manaus, João Pessoa, Recife, Rio de Janeiro, Belo Horizonte, Porto Alegre, Florianópolis, Campo Grande, and Cuiabá.

### Ethical considerations

The SMESH study was approved by the Research Ethics Committee (REC) of Hospital Moinhos de Vento and subsequently by the RECs of the co-participating centers, in accordance with the ethical principles established by Resolution 466/2012 of the Brazilian National Health Council^(12)^. This article presents the formative research conducted prior to data collection for the SMESH study. In this phase, an Informed Consent Form (ICF) was not required from participants, who included representatives of social movements, health managers, and healthcare professionals. However, all participants signed an ICF during the recruitment stage. Additional details about the study have been previously published^(13)^.

### Study design

This qualitative formative study was conducted as a preparatory stage of the RDS implementation. To ensure methodological rigor in reporting, the Standards for Reporting Qualitative Research (SRQR) were followed^(14)^.

### Methodological procedures

The formative research was supported by municipal health secretariats (SMS), which facilitated contact with members of social movements and non-governmental organizations (NGOs) who were invited to participate in in-person meetings with the study coordination team. These meetings followed three phases: (1) presentation of the study by the coordination team; (2) a roundtable discussion to clarify questions and explore potential adaptations to the local context; and (3) identification of seeds to initiate recruitment, suggested by social movement and NGO representatives.

### Study setting

The formative research meetings were held in locations such as university classrooms and meeting rooms made available by the municipal health secretariats in each capital. These spaces were designated specifically for this purpose to ensure confidentiality and privacy during the discussions.

### Data sources

The data sources included representatives of social movements and NGOs advocating for the rights of gay men/MSM and female sex workers, as well as healthcare professionals and health managers working in the participating capitals who could potentially serve as study site collaborators. Community involvement gives this work a co-productive character, which is essential to identifying the actual needs of these populations and reflects what has been increasingly encouraged in the scientific literature^(15)^.

### Data collection and organization

Studies using RDS commonly apply different data collection techniques during the formative phase, such as focus groups, interviews with key informants, and direct observation^(16)^. In the present study, in-person meetings were held between July 2018 and July 2021, and information was documented in reports specific to each city. These reports included content related to barriers and facilitators for recruitment, logistical aspects of data collection, engagement strategies, and methodological adaptations needed for the study. The timeline was extended due to the COVID-19 pandemic, which hindered the ability to travel for continued in-person meetings.

### Formative process phases

The formative research was structured into three phases. In the first phase, the study coordination team formally presented the project, including its objectives, rationale, data collection logistics, inclusion and exclusion criteria, planned tests, and topics covered in the questionnaire. In the second phase, a roundtable discussion was held, during which participants suggested more suitable locations and times for the research, discussed the type and value of incentives, proposed strategies for peer recruitment, provided input on the questionnaire’s language and content, expressed interest in receiving rapid tests for syphilis and HIV and the HPV test results, and identified potential challenges to study implementation. Finally, in the third phase, potential seeds for recruitment were identified, and coupons were distributed to those selected.

### Data analysis

Data analysis was based on the reading and synthesis of the reports produced in each city. The reports were analyzed collectively to identify the most relevant and recurring themes discussed during the meetings to inform the implementation of the study.

## RESULTS

A total of 11 meetings were held-one in each participating center, except in Porto Alegre, where two meetings were necessary. Healthcare professionals were the most represented group during the formative phase (n = 51), followed by representatives of lesbian, gay, bisexual, transgender, queer, intersex, and asexual (LGBTQIA+) social movements (n = 43). In some meetings, travestis, transgender, and transsexual individuals also participated after learning about the study through interactions with other social movements not directly included in the study population. As for the social movements representing sex workers, municipal health departments reported significant challenges in outreach and mobilization, which led to lower participation from this group (Table 1).

**Table 1:** Participants in the formative research by capital

City (state)	LGBTQIA+ representatives	Sex worker representatives	Health managers	Healthcare professionals
Belo Horizonte (MG)	3	1	1	8
Boa Vista (RR)	5	-	1	3
Campo Grande (MS)	6	-	1	7
Cuiabá (MT)	-	1	1	3
Florianópolis (SC)	6	-	1	2
João Pessoa (PB)	7	1	4	7
Manaus (AM)	5	1	3	7
Porto Alegre (RS)	5	2	-	5
Recife (PE)	2	-	3	5
Rio de Janeiro (RJ)	4	-	2	4
Total	43	6	17	51

*MG - Minas Gerais; RR - Roraima; MS - Mato Grosso do Sul; MT - Mato Grosso; SC - Santa Catarina; PB - Paraíba; AM - Amazonas; RS - Rio Grande do Sul; PE - Pernambuco; RJ - Rio de Janeiro; LGBTQIA+ - lesbian, gay, bisexual, travesti/transgender, queer, intersex, and asexual individuals.*

## DISCUSSION

### Logistical adaptation for data collection

The location for data collection was agreed upon by municipal health departments and social movements based on accessibility for the target populations and logistical feasibility for the departments. The type of healthcare service chosen for data collection was also adapted according to suggestions from social movements.

The initial plan to conduct data collection in primary healthcare units was revised to include Testing and Counseling Centers (CTAs - *Centros de Testagem e Aconselhamento*), as these are recognized as referral centers for healthcare services targeting these populations. Moreover, CTAs offer extended service hours to improve access for individuals who may be unavailable or uncomfortable accessing their usual healthcare units^(17)^. As a result, all regions-except the Northern Region-included at least one CTA as a data collection site.

Significant differences in engagement and reported needs between the two groups were identified during the formative research. For gay men and MSM, central areas of the city were preferred due to ease of transportation and proximity to gay social venues in most cities. In contrast, for sex workers, preferred areas were those located near prostitution zones.

Representation of the sex worker group was lower across all capitals, leading healthcare professionals, managers, and the study coordination team to anticipate greater challenges in recruiting this population. Healthcare professionals in every city reported difficulties in accessing this group, which they identified as a barrier to providing appropriate care. Several outreach strategies were proposed, such as visiting areas where sex work occurs to promote the study and inviting seeds from within the health unit’s catchment area. Similar challenges were encountered in the formative research conducted in Podgorica (Montenegro), characterized by low engagement from sex workers. They were not present in common areas of the city, and those found near brothels reported having restricted social networks and difficulty establishing bonds with peers. Moreover, they expected the study incentive to be equal to or higher than their earnings from a typical client^(18)^.

Data collection times were adjusted based on the needs of each group. Both identified evening hours as the most suitable, and for some gay men/MSM, it was the only feasible option. This group also suggested late afternoon as a possibility due to work or school commitments. Most sex workers stated that the best strategy would be to schedule appointments flexibly, depending on client demand, which posed significant logistical challenges for implementation. Collection times were coordinated with healthcare professionals, based on these preferences and service availability.

Regarding the study questionnaire, strategies to improve response rates include conducting interviews in private settings, establishing rapport with participants, and adapting the language to foster a sense of safety and comfort^(19)^. The formative research enabled adaptations to language and the development of a response card for sensitive questions, allowing participants to indicate their answers without speaking. Training sessions emphasized the importance of providing a welcoming, nonjudgmental environment so that participants would feel comfortable and willing to invite others to the study.

### Incentives e benefits

In RDS, participants receive an incentive for participation (primary incentive) and an additional amount for each peer recruited using coupons (secondary incentive)^(20)^. These incentives can be provided in the form of cash, meal vouchers, or giveaways. However, the amount is typically modest to avoid coercion^(13)^.

Due to operational constraints of the project, cash incentives were not available at the time of data collection. The initially planned payment method was a postal money order, later replaced by a prepaid card. This change was necessary to reduce the time for incentive delivery from 30 to 5 days. Nonetheless, social movement representatives identified the non-immediate availability of the incentive as a barrier to recruitment. In addition, some sex workers reported that a transportation voucher would be important to cover travel expenses.

The primary incentive amount set by the study coordination team was R$40.00 for participation and R$30.00 as a secondary incentive for each recruited peer. Satisfaction with the incentive value varied among sex workers, depending on the client fee charged for their services. Among gay men/MSM, the vast majority considered the amount adequate.

For this group, one of the main motivations was the offer of rapid tests for HIV and syphilis. In contrast, sex workers from one of the cities reported that rapid tests would not be a draw, as they were already part of their routine. Still, there was a consensus among representatives that this would be an opportunity for health promotion and disease prevention activities, including cytopathological screening. The availability of HPV testing was perceived as a key differentiator of the study, as this test is not available through the public health system. Delivering test results to participants, along with proper counseling and follow-up in cases of high-risk HPV diagnoses, was essential to the success of the study.

### Seed selection and peer recruitment

Selecting seeds with diverse characteristics supports effective recruitment and contributes to building a more representative sample of the population^(20,21)^. In this formative research, social movement representatives were informed of the importance of carefully selecting seeds who, although belonging to the same group (gay men/MSM or sex workers), had different profiles to ensure broader representativeness.

In each city, up to three seeds per group were selected to initiate the study. Each seed received three coupons to distribute to their peers. In most cases, at least one seed was identified during the formative phase. When social movements were not represented at the meetings, referrals were made later. It is understood that recruitment may vary depending on the target population; therefore, it is essential to understand each group’s needs to adapt mobilization and engagement strategies whenever possible^(22)^.

The MSM category includes individuals who engage in sexual activity with other men, regardless of whether they identify as homosexual^(23)^. According to LGBTQIA+ movement representatives from different locations, when individuals do not identify as gay or bisexual-even if they have sex with other men-recruitment may be more challenging.

A participant from the sex workers’ movement emphasized the importance of selecting seeds from different areas of the city, as there are regions marked by strong rivalries, making it unlikely that one group would hand out coupons to another. Moreover, including seeds from various city zones would help reflect the diversity within the sex worker population. In this group, it is also challenging to reach sex workers from higher socioeconomic classes, who do not identify as such but rather as “escorts”, distancing themselves from the identity and political mobilization of sex workers^(24)^. Additionally, the seasonal nature of sex work in tourist cities was identified as a recruitment barrier.

Most participants from social movements reported having broad and diverse social networks. As a result, the RDS strategy of distributing invitations was considered effective in recruiting individuals with different profiles. Furthermore, the WhatsApp messaging app was seen as a powerful tool for forming groups and facilitating communication between social movements and their peers.

A recurring concern raised by participants from some social movements during the meetings was data confidentiality, especially given that participants might belong to overlapping social networks. In this regard, the study coordination team reinforced its commitment to research ethics, presented the ICF, and assured participants of the confidentiality of the data.

### Study limitations

The main limitations of this study were related to the method of recording the meeting information, the low participation of sex workers, and the inability to implement all suggestions made by participants during the formative phase. The formative meetings were not audio-recorded but instead documented in written reports, which limited the depth of analysis of participants’ accounts. Sex workers showed low participation in the meetings, which may have led to less tailoring of the research tools, data collection sites, and times to this population’s specific needs. In most cases, consensus had to be reached, as there were divergent opinions among participants in the formative phase, and some suggestions were not feasible due to incompatibility with the study protocol or the constraints of local health services.

### Contributions to nursing and public health

The formative research phase is essential to create favorable conditions for implementing the RDS method, such as selecting appropriate seeds, identifying feasible data collection sites and times, understanding what motivates groups to participate and engage in the study, using appropriate language in the instruments, and designing logistics that are responsive to the realities of the target population-all of which enhance participant recruitment. In this sense, the study is highly relevant to nursing and public health, as it highlights both the importance of conducting formative research before the main study and the challenges involved, thus helping to strengthen future studies that adopt the RDS method.

## FINAL CONSIDERATIONS

The formative research phase is a key step in developing the RDS method. It enables the assessment of the population’s needs and facilitates local coordination to support the study’s implementation-fostering engagement and trust, which are essential when working with key populations. The study was well received by the social movements involved; however, some concerns were raised, such as the short timeframe for implementation and the difficulty in engaging sex workers to contribute to this phase.

Given the increasing use of the RDS method among key populations in Brazil, this study is expected to provide researchers with a broader understanding of both the opportunities and challenges involved in conducting formative research. While the findings are not generalizable, they may support future studies involving these populations.

Additionally, the limited participation of sex worker representatives in the meetings underscores the need to strengthen the relationship between the health sector and this group. Studies involving key populations in healthcare settings have the potential to foster greater engagement with the health system and to help dismantle access barriers and stigma in healthcare services.

## Data Availability

The research data are available only upon request.
