# Supplementary material for: Formative research to support the implementation of respondent-driven sampling in a national study on HPV among vulnerable populations
Source: Rev Bras Enferm. 2025 Dec 8;78(Suppl 4):e20240581. doi: 10.1590/0034-7167-2024-0581 (PMC12700575; doi:10.1590/0034-7167-2024-0581)
Supplement: Translation

# Translation: Pesquisa formativa para implementação do *Respondent-Driven Sampling* em estudo nacional sobre HPV em populações vulneráveis

**DOI:** 10.1590/0034-7167-2024-0581pt

**Authors:** Giovana Petracco de Miranda, Camila Bonalume Dall’ Aqua, Tássia Rolim Camargo, Natália Luiza Kops, Eliana Márcia Wendland

**Affiliations:** I Hospital Moinhos de Vento. Porto Alegre, Rio Grande do Sul, Brasil; II Universidade Federal de Ciências da Saúde de Porto Alegre. Porto Alegre, Rio Grande do Sul, Brasil

**Keywords:** Saúde Sexual, Minorias Sexuais e de Gênero, Populações Vulneráveis, Papilomavirus Humano, Método.

## Abstract

**Objetivos:**

descrever a pesquisa formativa realizada durante a implementação do método *Respondent Driven Sampling* para avaliar a prevalência de papilomavírus humano em *gays*, homens que fazem sexo com homens e trabalhadoras do sexo no Brasil.

**Métodos:**

foram realizados encontros entre a equipe do estudo, profissionais de saúde e representantes de movimentos sociais. Incluíram se duas capitais por região do Brasil entre 2018 e 2021, totalizando 11 encontros.

**Resultados:**

foram discutidos aspectos como adequação logística, definição de incentivos e benefícios, escolha das “sementes” e expectativas sobre o recrutamento.

**Considerações Finais:**

a pesquisa formativa é essencial para desenvolver estudos que usam o método *Respondent-Driven Sampling*, pois a participação dos representantes das populações-alvo auxilia na coleta de informações sobre suas especificidades.

## INTRODUÇÃO

De acordo com dados do *Global Burden of Disease* (GBD), 8,74 milhões de pessoas viviam com HIV no mundo em 1990^(1)^, com aumento significativo para 39,9 milhões em 2023^(2)^. A epidemia de HIV despertou um olhar diferenciado para alguns grupos no âmbito da saúde pública, em especial pessoas que estão em situações de vulnerabilidade e possuem um maior risco para algumas doenças devido aos determinantes sociais de saúde. Esses grupos passaram a ser conhecidos como populações chave ou populações vulneráveis. São eles: *gays* e outros homens que fazem sexo com homens (HSH), pessoas que usam álcool e outras drogas, trabalhadoras do sexo, pessoas transgênero e travestis, bem como pessoas privadas de liberdade^(3)^.

Essas populações são desproporcionalmente afetadas por algumas doenças específicas, especialmente infecções sexualmente transmissíveis (IST), em razão de barreiras de acesso à prevenção, testagem e tratamento^(3)^. Entre essas infecções, está o papilomavírus humano (HPV), que, além de causar lesões verrucosas, está associado ao desenvolvimento de diferentes tipos de câncer e é determinado pelos mesmos comportamentos e práticas que aumentam a vulnerabilidade a outras IST^(4)^.

Tais populações são reconhecidamente difíceis de serem acessadas, portanto requerem o uso de métodos específicos de amostragem para pesquisas científicas. Um desses métodos é o *Respondent-Driven Sampling* (RDS), criado nos anos 1990 com base na técnica bola de neve^(5)^. Algumas características importantes do RDS incluem a seleção de “sementes” - pessoas com boa rede de contatos que iniciam o estudo e o recrutamento por pares, no qual cada participante recebe cupons para convidar outras pessoas da mesma população. Por sua eficácia e capacidade de gerar estimativas ajustadas e generalizáveis, o RDS tornou-se popular e vem sendo amplamente utilizado por diversos estudos no Brasil^(6,7)^ e em outros países^(8-11)^.

Para que o RDS seja implementado com sucesso, é preciso realizar uma pesquisa formativa para coletar informações sobre a população e suas especificidades, além de inclui-la na organização e planejamento local do estudo. A pesquisa formativa antecede o início da coleta de dados do estudo. É uma etapa fundamental para adequações que contemplem as necessidades da população, garantindo seu envolvimento e facilitando o desenvolvimento do estudo.

## OBJETIVOS

Descrever a pesquisa formativa realizada durante a implementação do método *Respondent Driven Sampling* para avaliar a prevalência de papilomavírus humano em *gays*, homens que fazem sexo com homens e trabalhadoras do sexo no Brasil.

## MÉTODOS

Este estudo buscou compreender a organização social de *gays*/HSH e trabalhadoras do sexo nas capitais incluídas do estudo SMESH, para subsidiar a implementação do método RDS. O SMESH é um estudo transversal de abrangência nacional para avaliar a prevalência de HPV e outras IST em *gays*/HSH e trabalhadoras do sexo, de ambos os sexos biológicos. Dez capitais, contemplando as cinco regiões brasileiras, foram incluídas: Boa Vista, Manaus, João Pessoa, Recife, Rio de Janeiro, Belo Horizonte, Porto Alegre, Florianópolis, Campo Grande e Cuiabá.

### Aspectos éticos

O estudo SMESH foi aprovado pelo Comitê de Ética em Pesquisa (CEP) do Hospital Moinhos de Vento e, posteriormente, nos respectivos CEPs dos centros coparticipantes, atendendo aos preceitos éticos propostos pela Resolução 466/2012 do Conselho Nacional de Saúde^(12)^. O presente trabalho apresenta a pesquisa formativa que antecedeu a coleta de dados do estudo SMESH. Nesse contexto, o Termo de Consentimento Livre e Esclarecido (TCLE) não é exigido para os integrantes desta etapa, que incluem representantes de movimentos sociais, gestores e profissionais de saúde. No entanto, durante o recrutamento dos participantes, todos assinaram um TCLE. Informações adicionais sobre o estudo foram previamente publicadas^(13)^.

### Tipo de estudo

Trata-se de uma pesquisa qualitativa de caráter formativo, desenvolvida como etapa inerente à implementação do método RDS. A fim de garantir a observância dos critérios de rigor metodológico para redação da pesquisa, utilizou-se o *Standards for Reporting Qualitative Research* (SRQR)^(14)^.

### Procedimentos metodológicos

A pesquisa formativa contou com o apoio das secretarias municipais de saúde (SMS) para o contato com os membros de movimentos sociais e organizações não governamentais (ONG), que foram convidados a participar de encontros presenciais com a equipe de coordenação do estudo. Esses encontros seguiram três etapas: 1) Apresentação do estudo pela coordenação do projeto; 2) Roda de conversa para o esclarecimento de dúvidas e discussão de possíveis adaptações do estudo para a realidade local; e 3) Indicação de “sementes” para o início do recrutamento, feita pelos representantes dos movimentos sociais e ONGs.

### Cenário do estudo

Os encontros da pesquisa formativa ocorreram em espaços como salas de aula em universidades e salas de reuniões disponibilizadas pelas SMS de cada capital. Foram reservadas especificamente para esse fim, garantindo o sigilo e a privacidade das discussões.

### Fonte de dados

As fontes de dados foram representantes de movimentos sociais e de ONGs voltadas para a defesa dos direitos de *gays*/HSH e trabalhadoras do sexo, bem como profissionais e gestores da área da saúde atuantes nas capitais participantes, que possivelmente acolheriam o estudo em suas unidades de saúde. A participação da comunidade confere a este trabalho o caráter de coprodução, que se faz necessário para evidenciar as reais necessidades dessas populações e corrobora o que vem sendo incentivado no meio científico^(15)^.

### Coleta e organização dos dados

Estudos que utilizam o RDS frequentemente aplicam diferentes técnicas de coleta de dados na fase formativa, tais como grupos focais, entrevistas com informantes-chave e observação direta^(16)^. No presente estudo, os encontros presenciais foram realizados entre julho de 2018 e julho de 2021, com registros documentados em relatórios específicos por capital. Cada relatório documentou informações relacionadas a barreiras e facilitadores para o recrutamento, aspectos logísticos da coleta de dados, estratégias de engajamento e necessidades de adaptações metodológicas ao estudo. O período se prolongou devido à pandemia de COVID-19, que comprometeu a possibilidade de viagens para a continuidade dos encontros presenciais.

### Etapas do trabalho

A pesquisa formativa foi estruturada em três etapas. Na primeira, a equipe de coordenação do estudo apresentou formalmente o projeto, contemplando seus objetivos, justificativa, logística de coleta de dados, critérios de inclusão e exclusão, exames a serem coletados e temas abordados no questionário. Na segunda etapa, ocorreu uma roda de conversa, na qual os participantes puderam sugerir locais e horários mais adequados para a realização da pesquisa, discutir o tipo e o valor dos incentivos, propor estratégias para o recrutamento por pares, oferecer considerações sobre a linguagem e o conteúdo do questionário, manifestar interesse na realização de testes rápidos para sífilis e HIV e no resultado do exame de HPV e compartilhar potenciais dificuldades para a execução do estudo. Por fim, na terceira etapa, identificaram-se possíveis sementes para o início do recrutamento, com a distribuição de cupons para os indicados.

### Análise de dados

A análise dos dados foi realizada com base na leitura e sistematização dos relatórios produzidos em cada capital. Os relatórios foram analisados em conjunto, buscando-se identificar as informações mais relevantes e recorrentes nos encontros para subsidiar a implementação do estudo.

## RESULTADOS

No total, foram realizados 11 encontros, sendo um em cada centro coparticipante, com exceção de Porto Alegre, onde foram necessários dois encontros. Os profissionais de saúde foram o grupo mais presente na construção da fase formativa (n = 51), seguidos dos movimentos sociais de lésbicas, *gays*, bissexuais, travestis/transexuais, *queer*, intersexuais e assexuais (LGBTQIA+) (n = 43). Em alguns encontros, também participaram travestis, transexuais e transgêneros que tiveram conhecimento do estudo por meio de articulações com outros movimentos sociais não pertencentes à população-alvo do estudo. Quanto aos movimentos sociais de trabalhadoras do sexo, as SMS relataram grande dificuldade de contato e mobilização, o que se refletiu em menor adesão desse grupo (Tabela 1).

**Tabela 1:** Integrantes da pesquisa formativa em cada capital

Município (estado)	Representantes LGBTQIA+	Representantes Trabalhadoras do sexo	Gestores	Profissionais da saúde
Belo Horizonte (MG)	3	1	1	8
Boa Vista (RR)	5	-	1	3
Campo Grande (MS)	6	-	1	7
Cuiabá (MT)	-	1	1	3
Florianópolis (SC)	6	-	1	2
João Pessoa (PB)	7	1	4	7
Manaus (AM)	5	1	3	7
Porto Alegre (RS)	5	2	-	5
Recife (PE)	2	-	3	5
Rio de Janeiro (RJ)	4	-	2	4
Total	43	6	17	51

*MG - Minas Gerais; RR - Roraima; MS - Mato Grosso do Sul; MT - Mato Grosso; SC - Santa Catarina; PB - Paraíba; AM - Amazonas; RS - Rio Grande do Sul; PE - Pernambuco; RJ - Rio de Janeiro; LGBTQIA+ - lésbicas, gays, bissexuais, travestis/transexuais, queer, intersexuais e assexuais.*

## DISCUSSÃO

### Adequação da logística para a coleta de dados

O local designado para a coleta de dados foi acordado entre as secretarias municipais de saúde e os movimentos sociais de acordo com a acessibilidade dessas populações e com a viabilidade logística por parte das secretarias. O tipo de serviço de saúde para a realização das coletas também foi adaptado conforme as sugestões dos movimentos sociais.

A intenção inicial de realizar a coleta em unidades de atenção primária foi adaptada para incluir os Centros de Testagem e Aconselhamento (CTA), já que estes são locais reconhecidos como centros de referência na atenção à saúde para essas populações. Além disso, os CTAs oferecem possibilidade de horários diferenciados de atendimento, visando ampliar o acesso de usuários que não tenham disponibilidade ou não se sintam à vontade em acessar suas unidades de referência^(17)^. Assim, com exceção da Região Norte, todas as demais regiões tiveram pelo menos um CTA como local de coleta.

Existiram diferenças importantes no engajamento e nas necessidades apontadas entre os dois grupos durante a pesquisa formativa. Para os *gays*/HSH, a região central foi indicada como preferencial por causa da facilidade de transporte e da proximidade a áreas de socialização *gay* na maioria das cidades. Já para as trabalhadoras do sexo, as áreas preferenciais foram as localizadas próximas às regiões de prostituição.

A representação do grupo de trabalhadoras do sexo foi menor em todas as capitais, levando os profissionais de saúde, gestores e equipe de coordenação do estudo a antecipar maior dificuldade no recrutamento de tal grupo. A dificuldade de acessar essa população foi relatada pelos profissionais de saúde de todas as capitais como um limitador da atenção à saúde. Diferentes estratégias de alcance foram sugeridas, como visitas a locais de prostituição para divulgar o estudo e convidar sementes dentro da área de atuação da unidade de saúde de referência. Dificuldades semelhantes foram encontradas na pesquisa formativa realizada em Podgorica (Montenegro), marcada pela baixa adesão de trabalhadoras do sexo. Estas não se encontravam em áreas comuns da cidade. Aquelas que se localizavam próximas a casas de prostituição relataram rede de contatos restrita e dificuldade em estabelecer vínculos com pessoas do mesmo grupo. Além disso, esperavam que o valor do incentivo da pesquisa fosse igual ou superior ao valor de seus programas^(18)^.

Os horários para a coleta de dados foram adaptados de acordo com as necessidades dos grupos. Ambos apontaram o período da noite como adequado, sendo para os *gays*/HSH, por vezes, o único horário possível. Ainda, esse grupo indicou o final da tarde como uma possibilidade devido aos turnos de trabalho e/ou estudo. Já a maioria das trabalhadoras do sexo referiu que a melhor estratégia seria poder agendar as coletas em qualquer dia, a depender do movimento de clientes, o que gerou desafios logísticos importantes para a operacionalização do estudo. Os horários de agendamento das coletas foram pactuados com os profissionais de saúde com base nessas características e disponibilidade dos serviços.

Quanto ao questionário do estudo, algumas estratégias para obter sucesso na captação de respostas são coletar os dados em um ambiente privado, estabelecer vínculo com o participante e adaptar a linguagem para que ele se sinta seguro e acolhido^(19)^. A pesquisa formativa possibilitou realizar adaptações de linguagem e elaborar um cartão de respostas para utilizar em perguntas consideradas sensíveis, nas quais o participante poderia apontar a opção de resposta sem verbalizar. Nos treinamentos, foi enfatizada com os profissionais de saúde a importância do acolhimento e de postura neutra, livre de julgamentos, para que os participantes se sentissem confortáveis e dispostos a convidar outras pessoas.

### Incentivos e benefícios

No RDS, os indivíduos recebem um incentivo pela participação (incentivo primário) e um valor adicional a cada recrutamento feito com os cupons (incentivo secundário)^(20)^. Esses incentivos podem ser feitos tanto na forma de dinheiro quanto de vale-refeição ou brindes. No entanto, o valor costuma ser modesto para evitar a coerção na participação^(13)^.

Devido a questões operacionais do projeto, o incentivo em espécie não foi disponibilizado no momento da coleta de dados. A forma de repasse inicialmente estabelecida foi o vale postal dos Correios, posteriormente alterado para um cartão pré-pago. A alteração foi necessária em decorrência do tempo para disponibilização do incentivo, que passou de 30 para 5 dias. De todo modo, a disponibilização não imediata do valor foi apontada pelos movimentos sociais como um aspecto dificultador do recrutamento. Ainda, algumas trabalhadoras do sexo referiram que seria importante disponibilizar um vale-transporte além do incentivo, para evitar o custo de deslocamento.

O valor do incentivo primário estipulado pela equipe de coordenação do projeto foi de R$ 40,00 pela participação e de R$ 30,00 como incentivo secundário por cada convidado incluído. A satisfação com o valor do incentivo variou entre as trabalhadoras do sexo, dependendo do valor cobrado por programa. Entre os *gays*/HSH, a grande maioria considerou adequado o valor.

O grupo de *gays*/HSH teve como uma das principais motivações a oferta de testes rápidos para HIV e sífilis. Em contrapartida, trabalhadoras do sexo de uma das cidades relataram que a realização de testes rápidos não seria um atrativo por já fazerem parte de sua rotina. Ainda assim, foi consenso entre os representantes de que esta seria uma oportunidade para atividades de promoção de saúde e prevenção de agravos, incluindo coleta de exame citopatológico. A oferta do teste de HPV foi percebida como um diferencial do estudo, tendo em vista que esse exame não é disponibilizado no sistema público de saúde. A entrega do resultado do teste para o participante bem como orientação e conduta adequadas diante de diagnósticos positivos para HPV de alto risco foram fundamentais para o sucesso do estudo.

### Escolha das sementes e recrutamento por pares

A seleção de sementes com características diversas favorece o recrutamento adequado e contribui para a formação de uma amostra mais representativa da população^(20,21)^. Nesta pesquisa formativa, portanto, foi explicitada aos movimentos sociais a importância de selecionar cuidadosamente as sementes para que, embora do mesmo grupo (*gays*/HSH ou trabalhadoras do sexo), tivessem características diferentes entre si, garantindo maior representatividade.

Em cada cidade, foram selecionadas até três sementes por grupo para iniciar o estudo. Cada semente recebeu três cupons para distribuir a seus pares. Na maioria das vezes, pelo menos uma semente foi distribuída durante a fase formativa. Quando os movimentos não estavam representados nos encontros, as indicações ocorreram posteriormente. Entende-se que o recrutamento pode variar conforme a população-alvo; por isso, é importante conhecer as necessidades de cada grupo para adaptar as estratégias de mobilização e engajamento dos indivíduos, sempre que possível^(22)^.

A categoria HSH inclui indivíduos que mantêm relações sexuais com outros homens, independentemente de se identificarem ou não como homossexuais^(23)^. De acordo com os representantes dos movimentos LGBTQIA+ dos diferentes locais, quando os indivíduos não se identificam como *gays* ou bissexuais, apesar de praticarem sexo com outros homens, o recrutamento poderia ser mais desafiador.

Uma participante do movimento de trabalhadoras do sexo citou a importância de selecionar sementes de diferentes territórios, pois existem áreas de grande “rivalidade”, e provavelmente um grupo não entregaria cupons para outros grupos. Além disso, incluir sementes de diferentes zonas da cidade contemplaria a diversidade de trabalhadoras. Nesse grupo populacional, também é difícil acessar trabalhadoras do sexo de classes sociais mais altas, que não se reconhecem como tal, e sim como “acompanhantes”, o que contribui para seu distanciamento da identidade e mobilização política das trabalhadoras sexuais^(24)^. Ainda, a sazonalidade da força de trabalho sexual em cidades turísticas foi um fator apontado como barreira para o recrutamento.

A maior parte dos participantes dos movimentos sociais relatou que possui ampla e diversificada rede de contatos. Logo, avaliou-se que a estratégia do RDS de entregar convites seria eficaz no recrutamento de indivíduos com características distintas. Ainda, o aplicativo de mensagens WhatsApp foi indicado como um potente instrumento para formar grupos e facilitar a comunicação entre os movimentos sociais e seus respectivos pares.

Uma preocupação frequente dos participantes dos movimentos sociais em alguns encontros foi a confidencialidade dos dados, dado que os participantes podem pertencer a redes sociais comuns. Nesse sentido, a equipe de coordenação do estudo reforçou o compromisso com a ética em pesquisa, apresentou o TCLE e assegurou a confidencialidade dos dados.

### Limitações do estudo

As principais limitações deste estudo relacionaram-se à forma de registro das informações dos encontros, baixa adesão das profissionais do sexo e impossibilidade de implementar todas as sugestões dos participantes da formativa. Os encontros da fase formativa não foram gravados, e sim apenas registrados em relatórios, o que dificultou uma análise mais aprofundada dos relatos. Houve uma baixa adesão do grupo de profissionais do sexo nos encontros, o que pode ter refletido uma menor adequação das especificidades dessa população nos instrumentos da pesquisa, locais e horários da realização da coleta de dados. Na maioria das vezes, foi preciso entrar em um consenso, já que existiram divergências entre os participantes da fase formativa, e determinadas sugestões não eram factíveis por não se adequarem ao protocolo do estudo ou às possibilidades dos serviços de saúde.

### Contribuições para a área da Enfermagem e Saúde

A pesquisa formativa é essencial para fornecer as condições adequadas para a implementação do método RDS, tais como a escolha das sementes, indicações de locais e horários para a coleta de dados, entendimento da motivação dos grupos em participar e se engajar no estudo, utilização de linguagem apropriada nos instrumentos e promoção de uma logística sensível à realidade da população-alvo, favorecendo a participação efetiva dos recrutados. Desse modo, acredita-se que este trabalho é de grande relevância para a enfermagem e saúde, pois apresenta a importância da realização da pesquisa formativa (i.e., previamente ao estudo) e também as dificuldades envolvidas, possibilitando o aperfeiçoamento de futuras pesquisas que utilizem o RDS.

## CONSIDERAÇÕES FINAIS

A pesquisa formativa é etapa essencial para desenvolver o RDS. Por meio dela, podem se analisar as necessidades da população e oportunizar a organização local para realizar o estudo, gerando engajamento e confiança, essenciais para trabalhar com populações-chave. A proposta do estudo teve boa aceitação pelos movimentos sociais presentes. Porém, alguns pontos de atenção foram citados, como o curto prazo de execução do estudo e a dificuldade de acessar trabalhadoras do sexo para auxiliar na construção dessa etapa.

Considerando a crescente difusão do método RDS entre populações-chave no Brasil, espera se que este estudo proporcione para pesquisadores um cenário de possibilidades e desafios na execução das pesquisas formativas. Sabe-se que esses resultados não são generalizáveis, todavia podem dar suporte a futuras pesquisas com essas populações.

Além disso, a menor adesão de representantes de trabalhadoras do sexo nos encontros reflete a necessidade de uma aproximação da área da saúde com esse grupo. Pesquisas envolvendo populações-chave em serviços de saúde podem ter potencial para proporcionar aproximação desses usuários à rede de saúde, com desconstrução de barreiras de acesso e estigmas nos serviços de saúde.

## Data Availability

Os dados de pesquisa só estão disponíveis mediante solicitação.
